# Gastric Perforation Following Dog Bite in a Child

**Published:** 2015-09-01

**Authors:** Ashrarur Rahman Mitul, Khalid Mahmud

**Affiliations:** Department of Pediatric Surgery, Bangladesh Institute of Child Health and Dhaka Shishu (Children) Hospital, Bangladesh

**Keywords:** Gastric perforation, Dog bite, Children

## Abstract

Gastric perforation following dog bite is exceedingly rare event in pediatric population that requires emergency surgery. We report a 26 month old male who presented 36 hours after a dog bite over abdomen with pneumoperitoneum. At laparotomy, two perforations were found on the anterior surface of the stomach. The perforations were repaired primarily. The child made an uneventful postoperative recovery.

## INTRODUCTION

Pediatric dog bite injuries are common globally and may cause significant morbidities.[1,2] Commonly the face and skull are injured in children under seven year of age. Visceral injuries because of dog bites are very rare.[3] One such incidence is reported.

## CASE REPORT

A 26 month old child hailing from the suburb, presented with abdominal distention and bilious vomiting for 24 hours. The child suffered stray dog bite over the abdomen about 36 hours earlier while playing in the yard of their house. The dog reportedly had bitten several other persons in the territory before it was killed by the locals. The child was immediately taken to the Infectious Diseases Hospital where local wound cleansing was done, anti-rabies prophylaxis and tetanus toxoids were administered and the child was sent home as there were no immediate features of internal visceral injury. After 12 hours of the injury, the abdomen of the child started to distend, and there were several episodes of bilious vomiting.

On presentation, the boy was irritable, apprehensive, moderately dehydrated, with tachypnea, and tachycardia and febrile. Abdomen was distended prominently over the epigastrium, bowel sounds were absent and upper border of liver dullness was obliterated. There were two penetrating injury marks of the bite over the epigastrium and left hypochondrium four centimeters apart without any evisceration or oozing of blood or peritoneal fluid (Fig 1).

**Figure:**
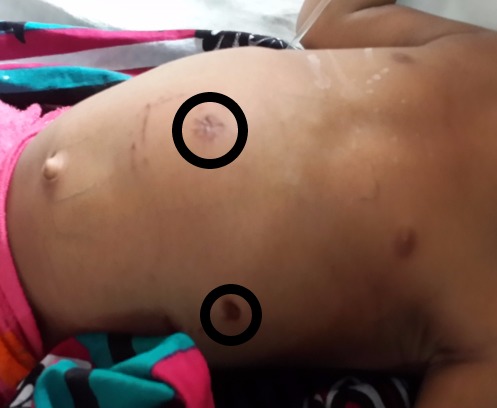
Figure 1:Marks of dog bite on anterior abdominal wall.

Investigations revealed Hb- 8.9gm/dl; with neutrophilic leukocytosis (TC of WBC: 13000/mm3; N-85%) and CRP was raised (70 mg/L). Plain x-ray abdomen revealed pneumoperitoneum. After initial resuscitation, he was operated and two small perforations (3cm apart) were found on the anterior wall of the stomach (Fig 2). The perforations were repaired after refreshing margins. Oral feed was initiated on the 4th postoperative day (POD). The boy made uneventful recovery. On first follow up after two weeks, the boy was found to be in good health with all wounds healed.

**Figure:**
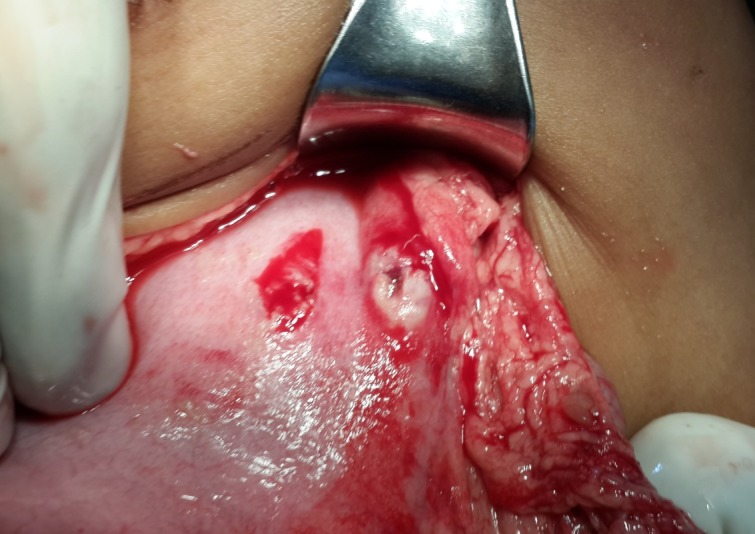
Figure 2:Two perforations on anterior wall of stomach

## DISCUSSION

Pediatric dog bite injuries are common and severity may vary from superficial wounds to life threatening injuries.[4] Even fatalities have been reported following dog bites.[5] In the developed countries, dog bites are most common cause of such injuries. Children due to their small size are more vulnerable to such injuries.[3, 6] There are reports of bowel evisceration with contusion of bowel with serosal tear from Nigeria. [7]

Visceral injuries following dog bite have been rare. A case with ileal injury following dog bite has been reported.[8] Another case of prolapsed rectum which was eaten up by a dog has been reported by Gopal SC et al.[9] Similar to the our case Baeza-Herrera C et al reported a patient who had two perforations on the anterior surface of the stomach.[3] Unlike their case, our patient did not need a gastrostomy. Though rare, dog bites can cause gastrointestinal injuries including perforations and the scenario has to be borne in mind while managing patients with animal bites.

## Footnotes

**Source of Support:** Nil

**Conflict of Interest:** None declared

